# Determinants of place birth: a multinomial logistic regression and spatial analysis of the Ethiopian mini demographic and health survey data, 2019

**DOI:** 10.1186/s12884-022-04880-z

**Published:** 2022-07-08

**Authors:** Temesgen Worku Gudayu

**Affiliations:** Department of Clinical Midwifery, School of Midwifery, College of Medicine and Health Sciences, University of Gondar, Gondar, Ethiopia

**Keywords:** Home birth, Home delivery, Place of birth, Ethiopia

## Abstract

**Background:**

Maternal and neonatal health significantly improves when birth is attended at health institutions where there are quality services and skilled attendants. In contrary, home birth results in high rates of maternal and neonatal mortality. Thus, this study aimed to determine the spatial distribution of home birth and to identify determinants of place of birth in Ethiopia based on the recent national survey.

**Methods:**

Ethiopian mini-DHS-2019 data was used in this analysis. A weighted sample of 5423 mothers were included. While health facility was a reference, home and health post were used as comparison categories to identify determinants of place of birth in a survey multinomial logistic regression model. An adjusted relative risk ratio, marginal effect, and a corresponding 95% confidence interval and a *p*-value of < 0.05 were used to declare statistical significance. The Global Moran’s I analysis was done by using ArcMap 10.8 to evaluate the clustering of home birth. The prevalence of home birth was predicted by ordinary kriging interpolation. Then, scanning was done by SaTScan V.9.6 software to detect scanning windows with low or high rates of home birth.

**Result:**

Prevalence of home birth in Ethiopia was 52.19% (95% CI: 46.49 – 57.83). Whereas, only 2.99% (95% CI: 1.68 – 5.25) of mothers gave birth in the health posts. Bigger family size, family wealth, multiparity, none and fewer antenatal visits, and low coverage of cluster level 4 + antenatal visits were predictors of home birth. Also, home birth was clustered across enumeration areas and it was over 40% in most parts of the country with > 75% in the Somali region. SaTScan analysis detected most likely primary clusters in the Somali region and secondary clusters in the rest five regions of the country.

**Conclusion:**

Home birth is a common practice in Ethiopia. Among public health facilities, health posts are the least utilized institutions for labor and delivery care. Nationally, implementing the 2016 WHO’s recommendations on antenatal care for a positive pregnancy experience and providing quality antenatal and delivery care in public facilities by qualified providers and back-up systems in place could be supportive.

## Background

Maternal mortality is a worldwide public health concern and a global estimate indicated that 211 Maternal Mortality Ratios (MMR) pre-100,000live births occurred in 2017. Southern Asia and sub-Saharan Africa countries contributed about 86% of the global MMR [1]. Ethiopia is among countries with the highest maternal mortality and the national survey indicated that 412 MMR occurred in 2016 [2].

Globally, between 2003 and 2013, about 72% of all maternal deaths occurred mainly due to hemorrhage, hypertensive disorder, and sepsis [3]. Studies from sub-Saharan Africa countries showed that maternal mortality was largely attributed to direct obstetrics causes. In Tanzania, nearly 84% of maternal mortality between 2006 and 2015 was due to eclampsia, obstetric hemorrhage, and sepsis [4]. Similarly, hospital-based trend studies in Ethiopia revealed that hemorrhage, pregnancy-induced hypertension, and sepsis played a significant role in maternal mortality [5, 6].

Several interventions are in place to combat maternal mortality. From the three risk periods of maternal mortality namely antepartum, intrapartum and postpartum; antenatal coverage was significantly reduced antepartum mortality, and the presence of skilled attendants at childbirth dropped intrapartum and early postpartum mortality [7]. Amongst several planned interventions, the Ethiopian government proposed to achieve over 90% coverage of 4 + antenatal visits and delivery attended by skilled providers by 2019/20 [8]. However, the 2019 national report indicated that 43% of mothers received 4 + antenatal care and 48% gave birth in the health facilities [9].

A recent quantitative study done in Ethiopia revealed that giving birth at home is a common practice. Rural residence, distance to health facility, low antenatal care coverage, and economic status are common societal factors. Also, low education, not planning for place birth, and unknown due date [10–12] were significant predictors. Moreover, socio-cultural factors such as assuming labor and delivery as a natural process, presence of enjoyable rituals during and after delivery, perceived friendly care by traditional attendants, and unavailability, inaccessibility, and perceived poor quality of modern services were qualitatively extracted factors [13–15] for home delivery in Ethiopia.

In most studies conducted in the past ten years in Ethiopia, the place of birth was measured as home and health facility. The health posts, the one in the primary health care systems [16] in the country, in all previous studies were considered as health facilities that provide basic and comprehensive obstetrics care. However, compared to other health facilities, health posts are supposed to be unequipped with basic facilities and services to provide skilled and quality labor and delivery care. Hence, treating health posts as separate category in the multinomial approach could yield better estimates than binary. Also, the exclusion of visitors in this study could result in a robust estimate. So that, the findings of this study would inform policymakers to consider all public health institutions in the future plans in order to achieve local and global targets of attended births by skilled providers.

## Methods

### Study area

The mini-Ethiopian demographic and health survey (EDHS) was conducted in Ethiopia. The survey was a nationwide mini-survey and included all nine reginal and two city administration areas. Besides regions and city administrations, the country is further subdivided into 68 zones, 817 districts, and 16,253 kebeles (the lowest level of administration) administrative structures [9].

### Data source and sampling procedure

**Fig. 1:**
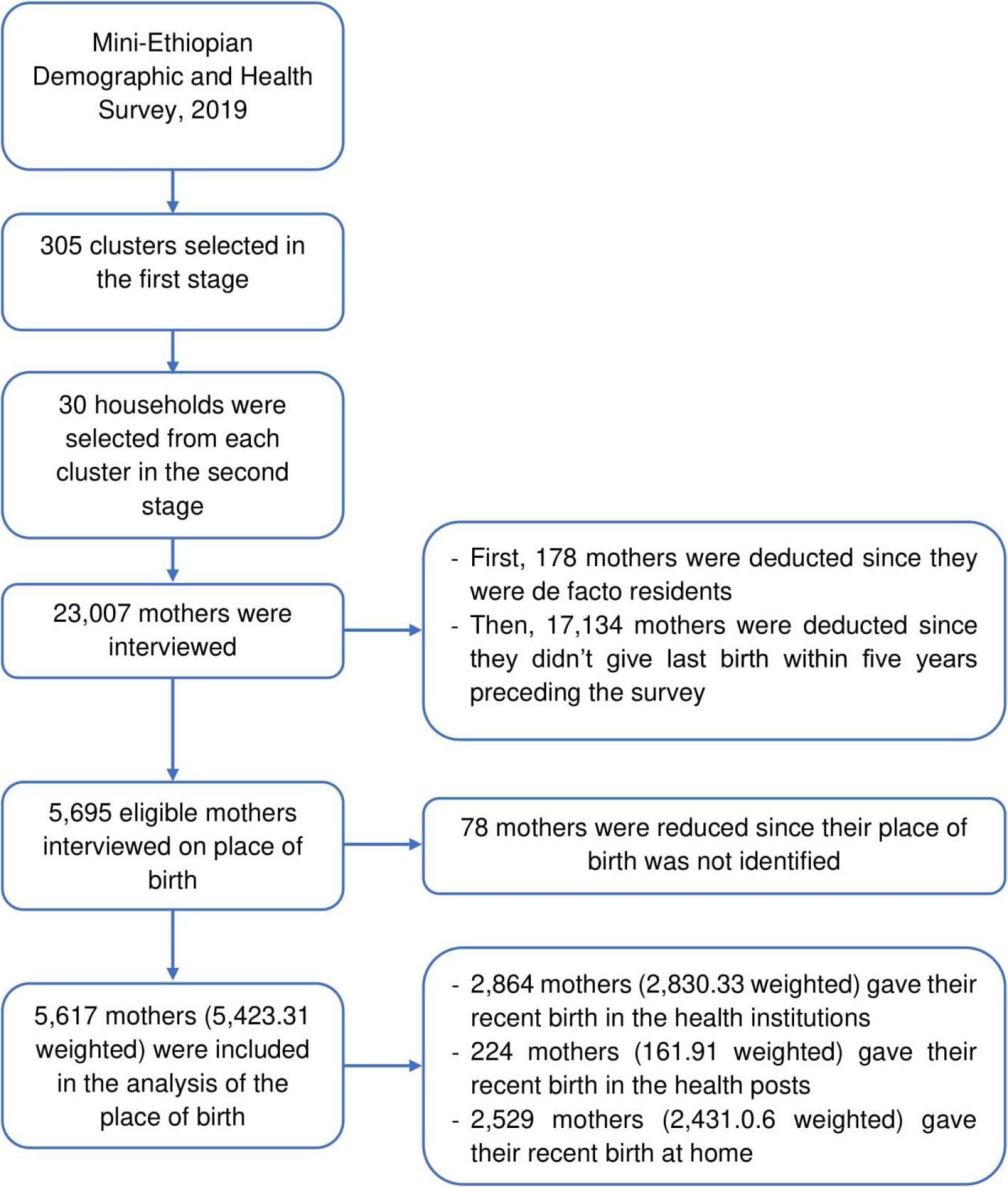
Flowchart of data extraction and sampling procedure, mini-Ethiopian demographic and health survey, 2019

The sampling frame used in the survey was the census enumeration areas (EAs) created for the upcoming Ethiopian Population and Housing Census (PHC). The EDHS is a nationally representative two-stage cluster cross-sectional survey. As described in detail in the EDHS 2019 report [9], in the first stage, 305 EAs (93 urban and 212 rural) were selected with probability proportional to EAs size and with independent selection of each sampling stratum (urban and rural). Then, in the second stage, 30 fixed households per cluster were selected with an equal probability systematic selection. In the current analysis, as shown in the figure (Fig. 1), a weighted total of 5423.31 mothers were included.

### Study variables

In this study, the outcome variable was the places of birth of the most recent child and places were categorized as (1 = home, 2 = health post, and 3 = health institution).

Health institutions are public hospitals and health centers, private hospitals and clinics, and nongovernmental organization (NGO) health facilities. These institutions are generally providing basic and comprehensive health services and labor and delivery care is usually provided by skilled providers.

Health posts: according to the three-tier health care delivery system of Ethiopia, are among the primary health care units and are satellite sites for health centers. Each health post is expected to serve a population of 3,000 – 5,000 and is a functional unit of health extension workers in rural areas [16]. These facilities are not equipped with skilled providers as per WHO’s definition [17]. Whereas, home in this study was the respondent’s home or other homes where a recent child birth took place.

The independent variables used in this analysis were both individual and community-level variables. Maternal age, media access, family size, maternal education attainment, family wealth index, parity, and antenatal care utilization were among individual-level variables. Whereas, place of residence, poverty level of the community, media accessibility of the community, literacy level of the community, and ≥ 4 antenatal care coverage at the community/cluster level were community-level variables included in the analysis.

Community-level variables such as poverty, media access, literacy, and cluster-level ≥ 4 antenatal care coverage were generated by aggregating individual-level variables at the community (cluster) level. Poorest and poorer family income categories were re-categorized as ‘poor’; maternal education category of no education was categorized as ‘illiteracy’; and family who didn’t access television or radio or both television and radio was categorized as ‘no’. Then, the prevalence of these variables was divided by the cluster size, and the generated value was further categorized as ‘low’ and ‘high’ based on the median value. Four and more antenatal care visits coverage was computed the same way but finally categorized in percentages as ‘below 25%’,’25–50%’, ‘51 – 74%’, and ‘ ≥ 75%’.

### Data analysis

#### Statistical analysis

Sociodemographic and reproductive characteristics of the study participants and the outcome variable were described in frequency and percentage.

A survey multinomial logistic regression model was used to analyze the association between the outcome and independent variables. Individual independent variables that had an association with place of birth at a *p*-value of < 0.2 were considered for the final multivariable model. The final survey multinomial multivariable model was selected based on the log likely (LL) ratio and the one with the highest LL ratio was selected. In the final model, an adjusted relative risk ratio (aRRR), its 95% confidence interval was computed. Also, marginal effect and its 95% confidence interval was calculated [18]. Then, the effect size, its corresponding interval, and a *p*-value of < 0.05 were interpreted and used to declare statistical significance.

#### Spatial analysis

The Global Moran’s I analysis was done by using ArcMap 10.8 to evaluate whether home birth is clustered, random, or dispersed across the study areas. Since home birth was clustered, spatial interpolation by using ArcMap 10.8 and scan statistics by using a SaTScan V.9.6 were carried out to predict the magnitude and to detect clusters and a scanning window with low or high rates of home birth.

## Result

Prevalence of place of birth, Socio-demographic, and reproductive characteristics of study participants.

The prevalence of home delivery in Ethiopia was 52.19% (95% CI: 46.49 – 57.83). Whereas delivery at health facilities was 44.83% (95% CI: 39.57 – 50.20) and only 2.99% (95% CI: 1.68 – 5.25) mothers gave birth in the health posts.

Nearly two-thirds (62.08%) of the mothers who didn’t expose access media in their household had given birth at home. Similarly, two-third and more mothers, whose family size was greater than six members were delivered at home. Giving birth at home showed a decreasing prevalence as mothers’ level of education and the family wealth index increases.

While most (86.34%) of the mothers who hadn’t get antenatal care, gave birth at home. The majority (66.35%) of grand multiparous mothers similarly delivered at home. Almost one-third of urban and two-third of rural residents gave home birth for their most recent delivery. From the regions in Ethiopia, Afar and Somali were the most common home birth regions in the country (Table 1).

**Table 1:** Sociodemographic and reproductive characteristics of mothers’ cross-tabulated with the place of birth of last birth in Ethiopian, 2019

Variables	Place of birth		
	**Health facility Frequency (%)**	**Health post Frequency (%)**	**Home Frequency (%)**
**Maternal age**			
15—19 years	133.8 (51.11)	10.39 (3.97)	117.6 (44.93)
20—34 years	1809 (46.41)	109 (2.8)	1980 (50.79)
≥ 35 years	488.3 (38.63)	42.54 (3.37)	733.2 (58)
**Media access at the household level**			
No	1241 (34.57)	120 (3.34)	2229 (62.08)
Yes	1190 (64.9)	41.87 (2.28)	601.6 (32.81)
**Family size**			
1–5	1446 (57.91)	69.42 (2.78)	981.3 (39.31)
6—10	943 (34.35)	92.45 (3.37)	1710 (62.29)
> 10	42.52 (23.44)	.0483 (0.03)	138.8 (76.53)
**Maternal educational level**			
No education	872.5 (29.96)	91.58 (3.14)	1948 (66.9)
Primary education	1062 (55.27)	55.08 (2.87)	804.2 (41.86)
Secondary education	321.8 (80.39)	14.1 (3.52)	804.2 (16.09)
Higher education	174.9 (92.32)	1.157 (0.61)	13.41 (7.07)
**Family wealth index**			
Poorest	216.9 (16.62)	42.31 (3.24)	1046 (80.13)
Poorer	418 (35.49)	38.13 (3.24)	721.5 (61.24)
Middle	408.1 (40.13)	35.66 (3.51)	573.2 (56.37)
Richer	542.8 (58.26)	36.21 (3.89)	352.7 (37.85)
Richest	845.3 (85.19)	9.609 (0.97)	137.3 (13.84)
**Parity**			
I	558.3 (70.74)	25.51 (3.23)	205.4 (26.03)
II—IV	1237 (48.86)	64.73 (2.56)	205.4 (48.58)
≥ V	636.1 (30.25)	71.67 (3.41)	71.67 (66.35)
**ANC during the index pregnancy**			
No ANC	125.3 (12.59)	10.66 (1.07)	859.5 (86.34)
1—3 ANC visits	579.9 (48.43)	46.65 (3.9)	570.9 (47.68)
≥ 4 ANC visits	1158 (70.5)	420.7 (3.9)	420.7 (25.61)
**Residence**			
Urban	935.7 (69.34)	15.19 (1.13)	398.6 (29.54)
Rural	1495 (36.71)	146.7 (3.6)	2432 (59.69)
**Region**			
Tigray	2432 (70.88)	7.661 (2.1)	98.45 (27.02)
Afar	98.45 (25.74)	2.065 (2.41)	61.52 (71.85)
Amhara	535.7 (52.64)	22.3 (2.19)	459.8 (45.17)
Oromia	802.3 (36.98)	91.65 (4.22)	1276 (58.8)
Somali	94.64 (23.34)	0.4412 (0.11)	310.3 (76.55)
Ben Shangul	27.88 (44.65)	13.6 (21.78)	20.96 (33.57)
SNNPR^a^	498 (45.46)	22.48 (2.05)	574.9 (52.49)
Gambela	16.4 (67.95)	0.5951 (2.47)	7.143 (29.59)
Harari	10.05 (63.23)	0.1808 (01.14)	5.663 (35.63)
Dire Dawa	19.23 (66.14)	0 .9357 (3.22)	8.905 (30.64)
Addis Ababa	146.5 (95.53)	0 (0)	6.85 (4.47)

^a^Southern Nations, Nationalities, and People’s Region

Spatial distribution of home birth in Ethiopia.

**Fig. 2:**
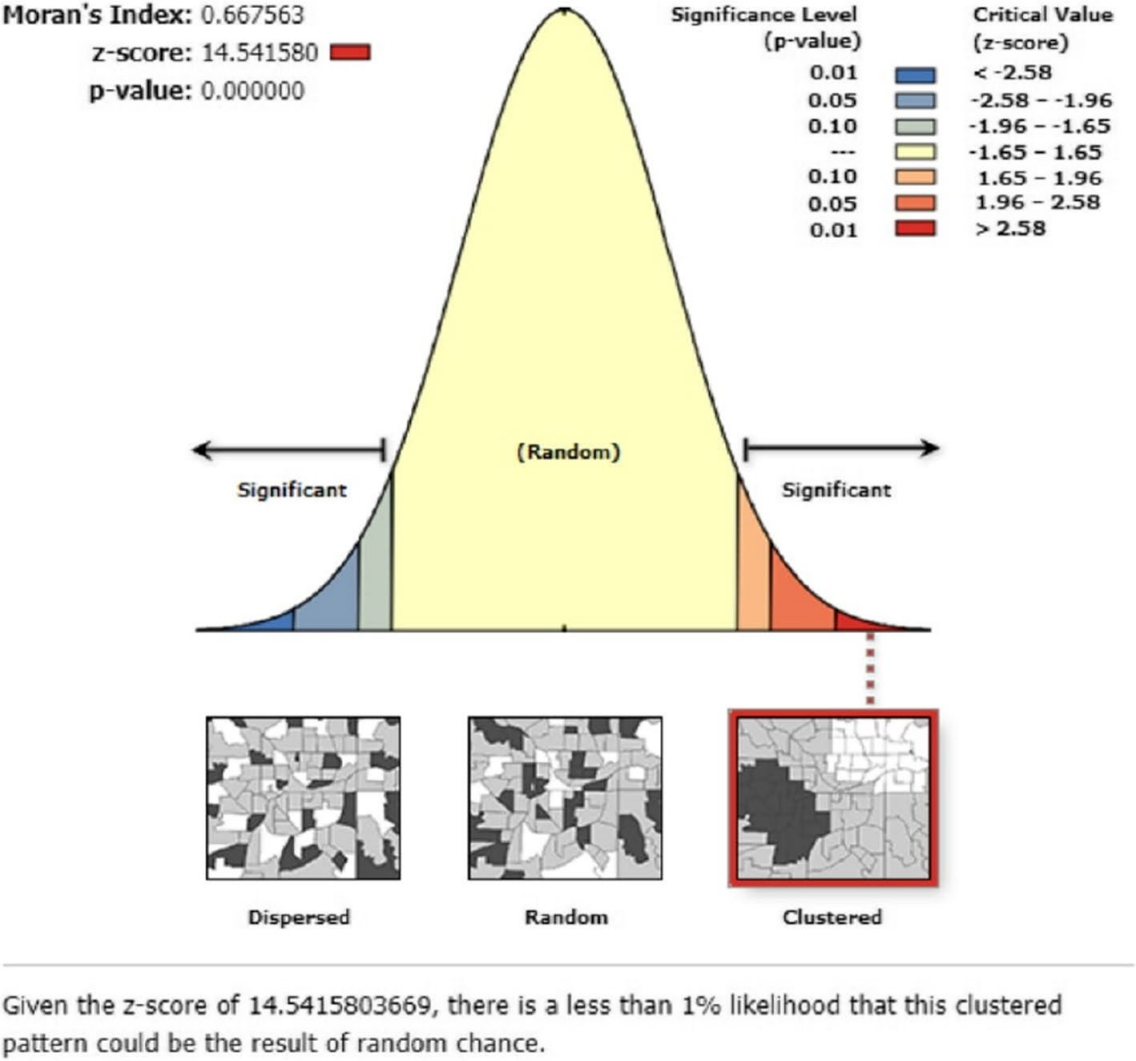
Global Moran’s I summary of spatial autocorrelation of home birth in Ethiopia, 2019

**Fig. 3:**
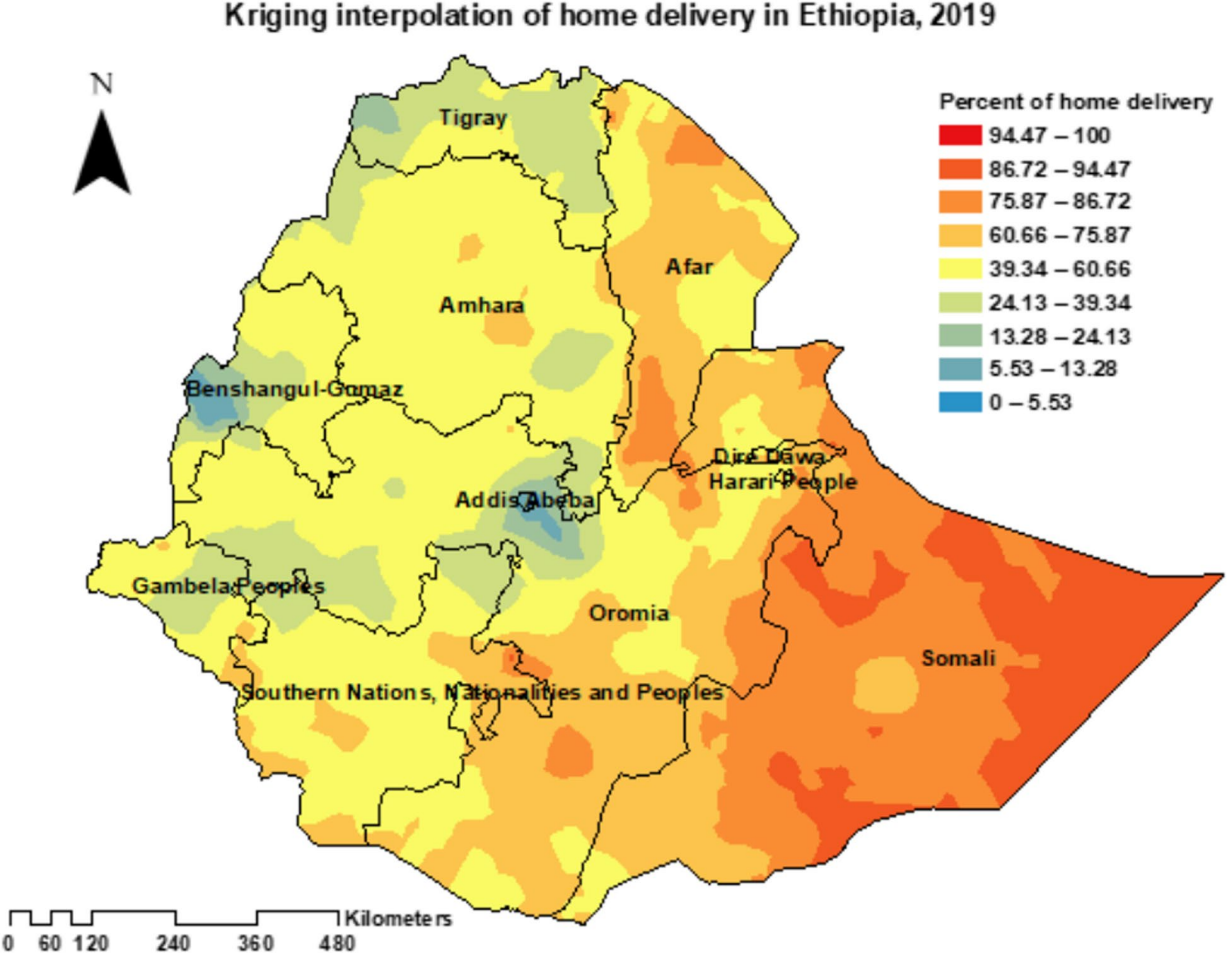
The kriging interpolation prediction of home birth in Ethiopia, 2019

A clustering pattern of home birth was revealed in the global spatial autocorrelation across the EAs (Moran’s index = 0.667563, z-score = 14.541580, *p*-value < 0.001) (Fig. 2). In addition, the ordinary kriging interpolation analysis predicted that home birth was relatively about 40% and higher in most parts of the country and more than 75% of home delivery was widely distributed in the Somali region (Fig. 3).

**Fig. 4:**
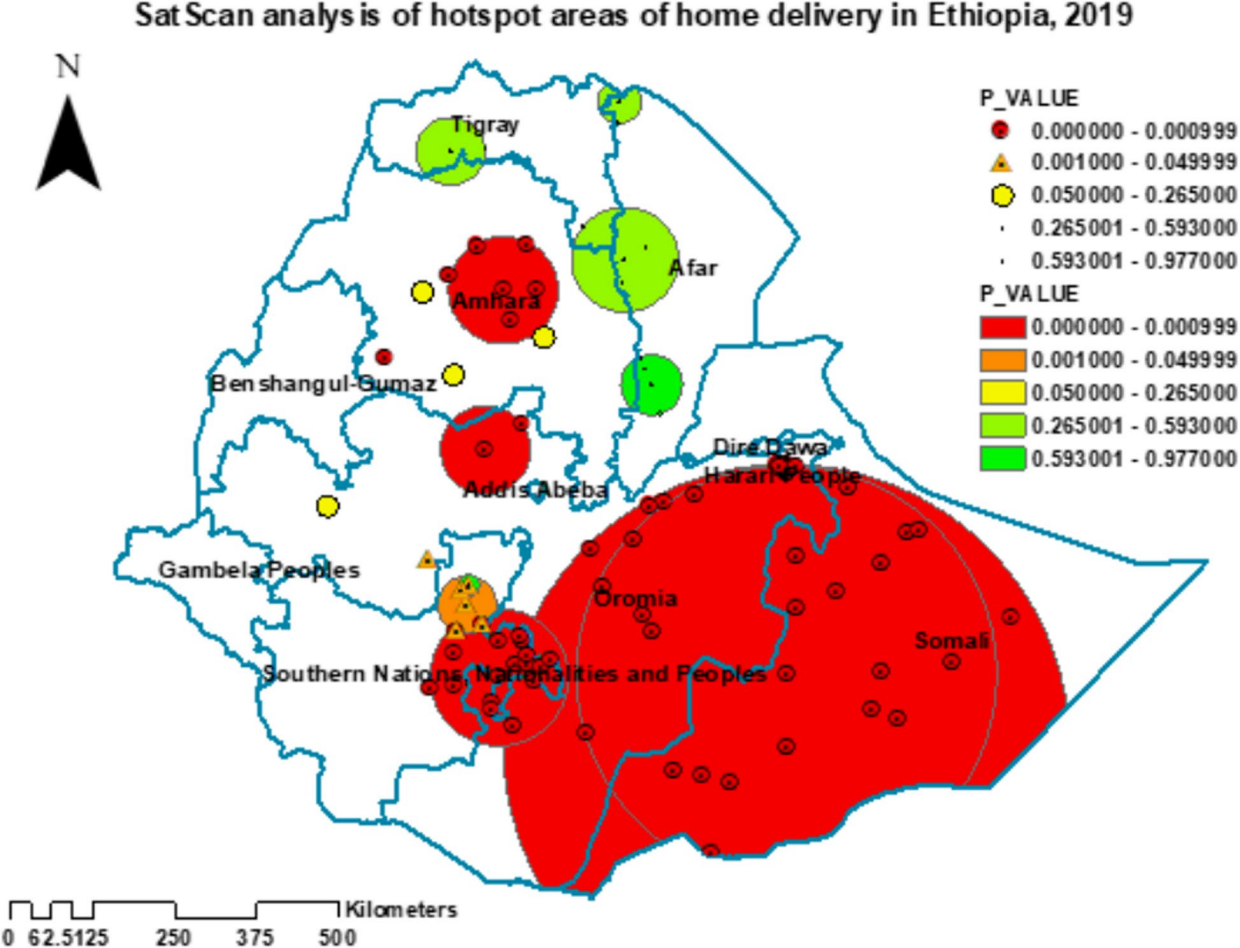
SaTScan hotspot analysis of home birth in Ethiopia, 2019

Also, the SaTScan analysis detected a total of seven statistically significant cluster areas with a high magnitude of home birth. The most likely primary cluster areas with the high prevalence of home birth were detected in the Somali region, Harari region, and eastern and southern zones of Oromia region with a relative risk (RR) = 1.72, and a *p*-value of < 0.001. In addition, the most likely secondary cluster areas with a high magnitude of home birth were spotted in the central zones of Amhara and eastern zones of South Nations, Nationalities, and People’s Region (SNNPR) (Fig. 4, Table 2).

**Table 2:** The most likely SaTScan clusters of areas with significant home birth in Ethiopia, 2019

**Location IDs**	**Region** (Zone [Woreda])	**Coordinate/ radius (Km)**	**Relative risk**	**LLR**	***p*-value**
142, 141, 136, 125, 138, 143, 137, 123, 144, 134, 145, 111, 135, 133, 110, 114, 131, 103, 122, 117, 132, 183, 102, 113, 140, 106, 129, 186, 88, 89, 181, 250, 105, 248, 104, 249, 244, 247, 255, 234, 233, 241, 243, 252, 245, 246, 237, 242, 235, 107, 231, 239, 240, 236, 254, 232, 182, 185	**Somali** (Fafan, Jarar, Nogob, Shabelle, Korahe, Doolo, Liben, Afder) **Oromia** (East and west Hararghe, East and west Arsi, Bale, Guji, Borena) **Hareri**	(5.479641 N, 42.196835 E) / 428.49 km	1.72	223.47	< 0.001
136, 134, 142, 138, 123, 145, 133, 137, 141, 125, 111, 131, 143, 110, 135, 122, 144, 132, 129, 106, 103, 250, 102, 248, 249, 114, 244, 247, 255, 234, 233, 241, 243, 252, 245, 246, 237, 242, 235, 107	**Somali** (Fafan, Jarar, Nogob, Shabelle, Korahe, Doolo, Liben, Afder) **Oromia** (East and west Hararghe, East and west Arsi, Bale, Guji, Borena) **Hareri**	(6.459193 N, 42.199432 E) / 317.51 km	1.77	200.76	< 0.001
115, 182, 172, 186, 188, 181, 113, 184, 185, 183, 197, 187, 190, 89, 117, 178, 189, 198	**Oromia** (West Arsi, Guji, Borena) **SNNPR** (Gedeo, Sidama, Wolayta, Gamo Gofa, Amaro)	(6.420265 N, 38.266739 E) / 104.43 km	1.54	100.72	< 0.001
80	**Ahara** (Agew Awi)	(10.779921 N, 36.711575 E) / 0 km	1.92	15.63	< 0.001
58, 60, 61, 83, 78, 57	**Amhara** (South Gondar, North Wollo, Wag Himra, North Gondar)	(11.722588 N, 38.322762 E) / 81.75 km	1.35	15.46	< 0.001
99, 100	**Oromia** (North and west Shewa)	(9.531226 N, 38.081684 E) / 67.38 km	1.56	13.99	< 0.001
180, 179, 177, 178, 189	**SNNPR** (Kembata Tembaro, Hadiya)	(7.415238 N, 37.827221 E) / 44.05 km	1.38	11.12	< 0.005

### Factors associated with place birth

The survey multinomial multivariable analysis identified that the relative probability of giving birth at home rather than health facility was about one and half times higher for mothers who had a family size of six to ten members than less than six members (aRRR = 1.46 (95% CI: 1.10, 1.93)). The marginal effect analysis also indicated that the probability of giving birth at home was on average five percentage (0.05 (0.01, 0.10)) points higher for mothers who had a family size of six to ten members. Whereas, the relative probability of giving birth at health post rather than health institution was 0.02 (aRRR = 0.02 (95% CI: 0.003, 0.20)) for mothers who had a family size greater than ten than less than six members implies that the probability of giving birth at health post on average three percentage (-0.03 (95% CI: -0.04, -0.009)) points lower for mothers who had largest family size than lowest family size.

The family wealth index was also found to be a predictor for a home birth. As compared to the richest family, the relative probability of giving birth at home rather than health facility among mothers was about two times higher for richer aRRR 2.13 (95% CI: 133., 3.43), more than four times higher for the middle (aRRR = 4.29 (95% CI: 2.68, 6.89)) and poorer (aRRR = 4.60 (95% CI: 2.70, 7.85)), and ten times higher for poorest (aRRR = 10.08 (95% CI: 5.66, 17.98)) family. As shown by the marginal effect analysis, the probability of giving birth at home among mothers was higher at 12 percentage points for richer, around 25 percentage points for middle and poorer families. Whereas, home birth was 38 percentage points higher for the poorest family.

In addition, the relative probability of giving birth at home rather than health facility was nearly twice higher for multiparous (aRRR = 1.95 (95% CI: 1.20, 3.15)) and grand multiparous (aRRR = 1.93 (95% CI: 1.08, 3.43)) mothers than primiparous mothers. The marginal effect analysis also revealed that the probability of giving birth at home was about ten percentage points higher among multiparous and grand multiparous than primiparous mothers.

Moreover, as compared to mothers who attended four and more antenatal care visits, the relative probability of giving birth at home rather than at a health facility was more than one times (aRRR = 1.59 (95% CI: 1.22, 2.07)) higher for mothers who attended less than four antenatal care visits and over six times (aRRR = 6.31 (95% CI: 4.27, 9.32)) higher for mothers who didn’t attend antenatal care during the index pregnancy. In the marginal effect analysis, the probability of giving birth at home was eight percentage points higher among mothers who attended less antenatal care visits and 30 percentage points higher among mothers who didn’t attend antenatal care than who attended four and more antenatal care visits. Likewise, the probability of giving birth at health posts on average two percentage (-0.02 (95% CI: -0.04, -0.001)) points lower for mothers who didn’t attend antenatal care.

Similarly, the relative probability of giving birth at home rather than health facility was more than two times (aRRR = 2.25 (95% CI: 1.20, 4.20)) higher among mothers who residing in the clusters in which 51—74% of mothers attended 4 + antenatal care visits than those residing in the clusters of ≥ 75% 4 + antenatal care visits coverage. And it was about five times (aRRR = 4.88 (95% CI: 2.40, 9.9)) higher among mothers who residing in the clusters in which 25 – 50% of mothers attended 4 + antenatal care visits and about seven times (aRRR = 749 (95% CI: 3.54, 15.85)) higher among mothers who residing in the clusters in which < 25% of mothers attended 4 + antenatal care visits as compared to those who residing in the clusters of ≥ 75% 4 + antenatal care visits coverage. The marginal analysis also showed that the probability of giving birth at home was 37 percentage points higher among mothers who residing in the clusters in which < 25% of mothers attended 4 + antenatal care visits, 30 percentage points higher among mothers who residing in the clusters in which 25 – 50% of mothers attended 4 + antenatal care vists, and 14 percentage points higher among mothers who residing in the clusters in which 51 – 74% of mothers attended 4 + antenatal care visits as compared to mothers who residing in the clusters in which ≥ 75% of mothers attended a coverage of 4 + antenatal care visits (Table 3).

**Table 3:** Survey multinomial multivariable analysis of factors associated with place of delivery in Ethiopia, 2019

Variables	Place of birth (the base outcome is Health facility)			
	**Home aRRR (95% CI)**	**Home Marginal effect (95% CI)**	**Health post aRRR (95% CI)**	**Health post Marginal effect (95% CI)**
**Maternal age**				
15—19 years	1	1	1	1
20—34 years	1.22 (0.66, 2.26)	0.04 (-0.06, 0.13)	0.69 (0.19, 2.57)	-0.02 (-0.07, 0.04)
≥ 35 years	0.88 (0.46, 1.69)	-0.01 (-0.11, 0.09)	0.54 (0.10, 2.82)	-0.02 (-0.08, 0.04)
**Media access at the household level**				
No	0.95 (0.69, 1.32)	-0.01 (-0.06, 0.04)	1.13 (0.54, 2.37)	0.004 (-0.01, 0.04)
Yes	1	1	1	1
**Family size**				
1–5	1	1	1	1
6—10	1.46 (1.10, 1.93) *	0.05 (0.01, 0.10) *	1.44 (0.85, 2.43)	0.005 (-0.009, 0.02)
> 10	1.61 (0.77, 3.36)	0.09 (-0.03, 0.19)	0.02 (0.003, 0.20) *	-0.03 (-0.04, -0.009) *
**Maternal educational level**				
No education	3.39 (0.84, 13.67)	0.19 (-0.04, 0.42)	3.48 (0.44, 27.39)	0.02 (-0.02, 0.05)
Primary education	1.92 (0.51, 7.28)	0.09 (-0.12, 0.31)	2.44 (0.31, 19.40)	0.01 (-0.02, 0.05)
Secondary education	1.16 (0.28, 4.89)	0.01 (-0.22, 0.24)	3.79 (0.38, 37.65)	0.04 (-0.03, 0.09)
Higher education	1	1	1	1
**Family wealth index**				
Poorest	10.08 (5.66, 17.98) **	0.38 (0.27, 0.49) **	7.72 (1.14, 52.22) *	0.03 (-0.02, 0.08)
Poorer	4.60 (2.70, 7.85) **	0.26 (0.16, 0.35) **	3.33 (0.60, 18.41)	0.01 (-0.02, 0.05)
Middle	4.29 (2.68, 6.89) **	0.25 (0.16, 0.34) **	2.99 (0.59, 15.16)	0.01 (-0.02, 0.04)
Richer	2.13 (1.33, 3.43) *	0.12 (0.04, 0.20) *	2.72 (0.59, 12.51)	0.02 (-0.01, 0.04)
Richest	1	1	1	1
**Parity**				
I	1	1	1	1
II—IV	1.95 (1.20, 3.15) *	0.10 (0.03, 0.18) *	1.21 (0.58, 2.51)	-0.002 (-0.03, 0.02)
≥ V	1.93 (1.08, 3.43) *	0.09 (0.008, 0.19) *	1.71 (0.66, 4.45)	0.008 (-0.02, 0.04)
**ANC during the index pregnancy**				
Data not available	1.93 (1.48, 2.52)	0.11 (0.07, 0.16)	1.08 (0.58, 2.00)	-0.007 (-0.02, 0.01)
No ANC	6.31 (4.27, 9.32) **	0.30 (0.24, 0.37) **	1.22 (0.39, 3.72)	-0.02 (-0.04, -0.001) *
1—3 ANC visits	1.59 (1.22, 2.07) **	0.08 (0.03, 0.12) *	1.24 (0.71, 2.17)	-0.0002 (-0.02, 0.02)
≥ 4 ANC visits	1	1	1	1
**Residence**				
Urban	1	1	1	1
Rural	0.78 (0.39, 1.59)	-0.05 (-0.15, 0.06)	2.91 (0.77, 11.02)	0.02 (-0.002, 0.05)
**The poverty level of the community**				
Low	1	1	1	1
High	0.79 (0.49, 1.26)	-0.04 (-0.11, 0.03)	1.47 (0.54, 4.04)	0.01 (-0.01, 0.04)
**Community media inaccessibility**				
Low	1	1	1	1
High	1.02 (0.69, 1.52)	0.01 (-0.05, 0.07)	0.64 (0.27, 1.53)	-0.01 (-0.04, 0.01)
**Illiteracy level of the community**				
Low	1	1	1	1
High	0.82 (0.56, 1.21)	-0.02 (-0.08, 0.03)	0.64 (0.28, 1.46)	-0.01 (-0.03, 0.01)
** ≥ 4 ANC use at the community level (cluster level coverage)**				
< 25%	7.49 (3.54, 15.85) **	0.37 (0.23, 0.51) **	0.46 (0.09, 2.39)	-0.05 (-0.12, 0.02)
25 – 50%	4.88 (2.40, 9.89) **	0.30 (0.16, 0.43) **	0.59 (0.17, 2.03)	-0.04 (-0.11, 0.02)
51 – 74%	2.25 (1.20, 4.20) *	0.14 (0.03, 0.26) *	1.17 (0.32, 4.33)	-0.01 (-0.08, 0.06)
≥ 75%	1	1	1	1

^**^p-value < 0.001, *p-value < 0.05

**Bold font:** Statistically significant variables and categories

## Discussion

In Ethiopia, the practice of giving birth at home showed a decreasing trend since 2005. From about 95% in 2005, home birth significantly dropped to 52% in 2019 [9]. This analysis revealed that 52.19% of mothers had given their most recent birth at home while 45% delivered at health facilities and only 3% delivered at health posts. The spatial analysis further identified that home birth in Ethiopia is clustered. Somali region and Harari regions, eastern and southern zones of Oromia region, eastern zones of SNNPR, and central zones of Amhara region were significant primary and secondary clusters of home birth in the five years preceding 2019 in Ethiopia. This finding is persistently similar to the 2011 and 2016 EDHS data-based analysis [12]. As explored by qualitative findings, home birth is common due to cultural reasons. Most society and women believe that labor and delivery is a natural process and the ritual processes during labor and delivery at home are pleasant [13]. Study participants further pointed out that mothers lack such joyful customs at health facilities [14].

Mothers from a family size above five members and those who were multiparous inclined to give birth at home than smaller family size and nulliparous mothers. Family size is directly related to birth order and similar studies also identified that higher birth order and multiparity were found to be significant factors for home birth [12]. Scientific explanations for the relation of parity and home birth are deficient. It could be explained by birthing experience, unpleasant experience from previous health facility birth, and cultural reasons as revealed by qualitative findings [13, 14, 19, 20].

This study further identified that antenatal care attendance at an individual level and its frequency, as well as community-level coverage of 4 + antenatal care, played a significant role in determining the place of birth. Several small- and large-scale studies also revealed that receiving no antenatal care [12] and delay in receiving antenatal care [21] were significantly associated with home delivery. Homebirth also significantly contributed by late entry to antenatal care [22] and receiving fewer than four antenatal care [23, 24] among antenatal care booked mothers. In countries like Ethiopia in which preconception care is not in place, antenatal care is an important entry for the continuum of maternity care. Pieces of evidence revealed that antenatal care when provided with a minimum recommended quality, it found to increase the likelihood of institutional delivery in developing countries [25, 26].

## Conclusion

In Ethiopia, home birth is a common practice. In contrast, health posts, which are community-level governmental health units, are the least utilized facilities for labor and delivery service. Individual-level none and fewer visits of antenatal care and lower cluster level coverage of 4 + antenatal care played a significant role in predicting home birth in Ethiopia. Nationally adapting the 2016 WHO’s recommendations on antenatal care for a positive pregnancy experience and providing quality antenatal and delivery care in public facilities by skilled provisers and systems of back-up in place could be helpful. Also, piloting the benefits of planned home birth with qualified professionals for low-risk pregnancies could be worth more.

## Data Availability

The datasets generated and/or analyzed during the current study are available in the [the DHS program] repository, [https://dhsprogram.com/].

## Abbreviations

ANC: Antenatal Care
aRRR: Adjusted Relative Risk Ratio
DHS: Demographic and Health Survey
EAs: Enumeration Areas
EDHS: Ethiopian Demographic and Health Survey
LL: Log Likely
MMR: Maternal Mortality Ratio
NGO: Non-Governmental Organizations
PHC: Population and Housing Census
